# Association between periodontitis and anti-citrullinated protein antibodies in rheumatoid arthritis patients: a cross-sectional study

**DOI:** 10.1186/s13075-020-2121-6

**Published:** 2020-02-13

**Authors:** Jerián González-Febles, Beatriz Rodríguez-Lozano, Carlos Sánchez-Piedra, Jorge Garnier-Rodríguez, Sagrario Bustabad, Martina Hernández-González, Enrique González-Dávila, Mariano Sanz, Federico Díaz-González

**Affiliations:** 1 Departamento de Especialidades Odontológicas, Facultad de Odontología, Universidad Complutense, Madrid, Spain; 2 Grupo de Investigación de Etiología y Tratamiento de las Enfermedades Periodontales (ETEP), Facultad de Odontología, Universidad Complutense, Madrid, Spain; 3 Servicio de Reumatología, Hospital Universitario de Canarias, S/C de Tenerife, Spain; 4 Unidad de Investigación de la Sociedad Española de Reumatología, Madrid, Spain; 5 Clínica Dental Dr. Garnier, S/C de Tenerife, Spain; 6 Departamento de Matemáticas, Estadística e Investigación Operativa, Universidad de La Laguna, San Cristóbal de La Laguna, Spain; 7 Departamento de Medicina Interna, Facultad de Medicina, Universidad de La Laguna, C/Ofra s/n, 38320 La Laguna, Spain

**Keywords:** ACPA, Rheumatoid factor, Severe periodontitis, Rheumatoid arthritis

## Abstract

**Aim:**

The aim of this study was to evaluate the association between periodontal parameters related with the periodontal disease severity and the presence and levels of anti-citrullinated protein antibodies (ACPAs) in rheumatoid arthritis (RA) patients.

**Materials and methods:**

This cross-sectional study included 164 RA patients. Socio-demographics and RA disease characteristics, including ELISA-detected ACPA (anti-CCP-2), were recorded. Exposure was assessed by periodontal parameters: plaque index (PI), bleeding on probing (BoP), probing pocket depth, and clinical attachment levels (CAL). Presence and levels of ACPAs (outcome) and exposure variables were compared by both parametric and non-parametric tests and associations were evaluated by adjusted odds ratio (OR).

**Results:**

A significant association was observed between the presence of anti-CCP antibodies and severity of periodontal outcomes such as the mean CAL (OR 1.483, *p* = 0.036), mean PI (OR 1.029, *p* = 0.012), and the number of pockets ≥ 5 mm (OR 1.021, *p* = 0.08). High anti-CCP antibodies levels were associated with mean CAL, mean PI, and number of pockets ≥ 5 mm with an OR of 1.593 (*p* = 0.043), 1.060 (*p* <  0.001), and 1.031 (*p* = 0.031), respectively. Furthermore, a significant increase of 4.45 U/mL in anti-CCP antibodies levels (*p* = 0.002) in RA patients was found for each pocket ≥ 5 mm after adjusting for age, gender, smoking, time of disease evolution, and RA activity.

**Conclusions:**

In RA patients, the severity of periodontal conditions such as mean CAL, mean PI, and the number of pockets ≥ 5 mm were linearly associated with both the presence and levels of anti-CCP antibodies.

## Key messages

1. Periodontitis severity parameters such as CAL and pockets ≥ 5 mm are associated with anti-CCP antibodies levels.
2. This association was more pronounced in patients with higher levels of these antibodies.
3. There is a linear correlation between periodontal parameters such as pockets ≥ 5 mm and anti-CCP titers.

## Introduction

Rheumatoid arthritis (RA) is a chronic systemic autoimmune disease characterized by painful joint inflammation, disability, and increased mortality [1]. Although knowledge of the pathogenesis underlying RA has increased substantially during the last decade, its etiology is still unknown. A complex interplay of genetic, environmental, and hormonal factors seem to influence the host immune tolerance leading to the characteristic autoimmune response of RA mainly characterized by the presence of rheumatoid factor (RF) and anti-citrullinated protein antibodies (ACPAs). These factors may also affect the mucosal surfaces of lungs, gut, and/or the periodontium [2, 3].

While citrullination, a post-translational protein modification caused by the enzyme peptidyl arginine deiminase (PAD), is not an exclusive process of RA, the formation of ACPAs is mainly restricted to RA patients. In fact, evidence strongly suggests that these antibodies are markers of more aggressive disease [4–6]. Although other factors may be able to induce protein citrullination [7], *Porphyromonas gingivalis* a key pathogen associated with the pathogenesis of periodontitis by inducing dysbiotic changes in the subgingival biofilm [8, 9] releases a specific deaminase, which has been linked to protein citrullination, thereby with potential to stimulate ACPA formation in RA patients [10, 11]. In fact, in patients with chronic periodontitis, high levels of citrullinated proteins have been identified within the periodontal tissues [12] and a recent in vivo experimental study has demonstrated a positive correlation between *P. gingivalis*-induced periodontitis and anti-cyclic citrullinated peptide (anti-CCP) antibodies levels in rats [13]. Furthermore, recently published evidence suggests that the presence of *P. gingivalis* infections may precede the clinical onset of RA years in advance [14].

Nowadays, there is solid evidence supporting an epidemiological association between periodontitis and RA [15–21]. Recently, our group has shown a significant and consistent association between these two diseases, mainly between severe periodontitis and RA disease activity [21]. Nonetheless, the relationship between *P. gingivalis*, periodontitis, and the presence of ACPAs has been a controversial topic. Some authors have remarked upon the specific role of *P. gingivalis* in shaping autoantibody specificity in RA patients [10, 22] independently of smoking status [10]. In fact, anti-CCP antibodies have been identified in periodontitis patients without RA [23, 24]. However, there are some studies that have not supported this association between periodontitis, the presence of *P. gingivalis*, and seropositivity for anti-CCP antibodies [10, 25, 26].

It was, therefore, the main objective of this cross-sectional study to further investigate the link between periodontitis and its severity with the presence and levels of anti-CCP antibodies in RA patients. In addition, the possible impact of shared risk factors as tobacco habit on the presence and levels of anti-CCP antibodies was evaluated.

## Methods

### Study population

Study subjects were consecutively included from those outpatients attending the Department of Rheumatology from January to September 2016. RA patients aged 18 years and older who fulfilled the 2010 ACR/EULAR classification criteria [27] were invited to participate. Patients with less than 8 teeth [17], who had received periodontal or antibiotic treatment during the previous 6 months [28], who had undergone joint replacement(s), who were in need of antibiotic prophylaxis, or who were being treated with cyclosporine A or anticonvulsants [29] were excluded. Patients treated with oral glucocorticoids maintained a stable dose during the month prior to their periodontal assessment and none received systemic treatment with these products during this period. RA patients who received intraarticular glucocorticoids during the month prior to evaluation were excluded from this study.

All enrolled patients were informed about the objectives and characteristics of the study and signed a written informed consent that had been previously approved by the Ethics Committee of the Hospital Universitario de Canarias (code 2015_069). Additionally, all the study recordings were performed in agreement with the principles of the Declaration of Helsinki.

### Study design

This was an observational, cross-sectional study of RA patients treated in a single Rheumatology Department who were assessed for the presence and severity of periodontitis.

#### Medical examination

All RA patients were subjected to a routine medical examination. Both the patient’s and care provider’s global assessments of disease activity were based on a 100-mm visual analog scale (VAS). Disease activity was calculated by means of 28-joint Disease Activity Score (DAS28), DAS28-C-reactive protein (CRP) [30], and Simplified Disease Activity Index (SDAI) [31] scores, and the medications used were logged from the medical files and by asking the patient during examination. Blood samples were tested for plasma RF and CRP, using an immunoturbidimetric assay (Roche/cobas® 8000 Modular Analyzer Series, Roche Diagnostics, USA), and for ACPA (anti-CCP-2) by Immunoscan CCPlus®. Euro Diagnostica with a positive value established as that exceeding 25 U/mL in both serological tests, and 3 mg/L in the CRP test. Anti-CCP antibody levels were stratified as low (between 25 and 75 U/mL), moderate (76–300 U/mL), or high (> 300 U/mL).

#### Periodontal examination

Full oral and periodontal examinations were carried out independently by two experienced periodontists. A kappa test showed 70% inter-examiner concordance. Full mouth probing pocket depth (PPD) and clinical attachment level (CAL) measurements were registered using an UNC-15 periodontal probe (six sites per tooth), excluding third molars and implants. Although subjects were informed of their periodontal status and advised to seek periodontal therapy when appropriate, no periodontal therapy was rendered as part of this investigation.

### Study variables

To evaluate RA, the following parameters were recorded: DAS28 using the erythrocyte sedimentation rate (ESR) (DAS28) or C-reactive protein (DAS28-CRP) [30], SDAI [31], RF, and ACPA presence and titers. Patients were categorized as being in remission, or having low, moderate, and high disease activity when at least two of the DAS28, DAS28-CRP, and SDAI scores were in agreement with the level of disease activity [21]. According to the time of disease evolution, patients with less than 2 years were classified as early RA.

Patient periodontal status was assessed using the following parameters: Full mouth plaque index (PI) and bleeding on probing (BoP) [32] were reported as mean percent of plaque and gingivitis, respectively [33], PPD, mean clinical attachment level (CAL), and tooth loss (dental implants and third molars were excluded). Based on these parameters, patients were categorized using two different case definition criteria for periodontitis: the 2005 Tonetti’s definition [34], which establishes 3 levels—level 0, individuals with a healthy periodontium; level 1, presence of proximal attachment loss ≥ 3 mm in ≥ 2 nonadjacent teeth; and level 2, presence of proximal attachment loss ≥ 5 mm in ≥ 30% of teeth—and the 2018 Tonetti’s definition [35], which grades periodontitis mainly based on the interdental CAL at the site of greatest loss and also on radiographic bone loss and on tooth loss, over 5 stages—stage 0, individuals with a healthy periodontium; stage I, initial; stage II, moderate; stage III, severe; and stage IV, advanced periodontitis.

The presences of co-morbidities, such as diabetes mellitus, osteoporosis, myocardial infarction, or dyslipidemia were recorded, as were anthropometric and socioeconomic variables. These included body mass index (BMI); smoking status (none, former and current); stress via the Perceived Stress Scale (PSS-14), categorized as high stress: yes (> 28 points) or no (≤ 28 points) [36, 37]; and social welfare indicators using the Graffar Scale Questionnaire [38]. History of therapy involving glucocorticoids, synthetic disease-modifying antirheumatic drugs (sDMARDs), and biologic DMARDs (bDMARDs) were also recorded.

### Statistical analyses

Descriptive data are presented as means, standard deviations (SD), and frequency distributions. *t* test and one-way ANOVA were used in continuous variables to analyze inter-group comparisons. Chi-square tests were used to compare categorical variables and when expected values were less than 5; the Fisher’s exact test in 2 × 2 tables was used.

To study the association between the presence of periodontitis (exposure) and anti-CCP antibody presence (outcome), a logistic regression model was constructed calculating the odds by means of odds ratios (OR), and 95% confidence intervals. Each analysis was adjusted for possible confounders (covariates) such as age, sex, tobacco use, time of disease evolution, and disease activity.

In addition, a logistic regression model with the backward Wald variable selection method was used to analyze the influence of periodontal variables over the presence of anti-CCP antibody, adjusting for the same covariates. Hence, ROC (receiver operating characteristic) curves and the areas under those curves (AUC-ROC) were analyzed.

In order to investigate the relationship between periodontal parameters as mean PI (in %), number of pocket ≥ 5 mm, BoP, and mean CAL (exposure) and anti-CCP antibody levels (multinomial outcome), an ordinal logistic regression model adjusted by age, sex, time of RA evolution, and RA disease activity and tobacco was carried out. Afterwards, an analysis of the covariates (ANCOVA) was performed to elucidate the association between anti-CCP antibody quantification and the number of pockets ≥ 5 mm, controlling for the same confounders.

Finally, adjusted OR calculations were carried out to determine the association between tobacco consumption and periodontitis. An ordinal logistic regression model was applied to assess the relationship between periodontitis and tobacco consumption (exposures) and anti-CCP antibody levels (outcome), including the interactions between them. The statistical analyses were performed using the statistical package SPSS 25 (IBM SPSS, Armonk, NY).

## Results

### Characteristics of RA patients

In total, 164 RA patients, 77% women, with a mean age of 54.1 ± 10.46 years and mean disease duration of 8.3 ± 7.23 years were included in this study. Positivity for anti-CCP antibodies (> 25 UI/ml) was detected in 109 patients (66.5%), being distributed according to the antibody level titers in low (28%), moderate (42%), and high (30%). Demographic characteristics and the presence of co-morbidities in this RA population are shown in Table 1, being described for the total RA patient population, and those with presence or absence of anti-CCP antibodies. Presence of anti-CCP antibodies was not associated with age, gender, race, CRP, BMD, smoking, dyslipidemia, hypertension, BMI, or myocardial infarction, while a significant association was demonstrated with higher stress levels, ESR, and overweight. There was a tendency for an association between presence of anti-CCP antibodies and reduced attendance to oral preventive interventions and lower socioeconomic status.

**Table 1:** Demographic and anthropometric characteristics and co-morbidities of RA patients

	Anti-CCP negative (*N* = 55)	Anti-CCP positive (*N* = 109)	*p* value	Total (*N* = 164)
Gender			0.815	
Female	42 (76%)	85 (78%)		127 (77%)
Male	13 (24%)	24 (22%)		37 (23%)
Ratio F/M				3.4
Mean age (SD) years	54.9 (9.79)	53.7 (10.81)	0.490	54.1 (10.46)
Race				
Caucasian	54 (98%)	105 (96%)	0.723	159 (97%)
Graffar			0.064	
High	1 (2%)	10 (9%)		11 (6%)
Medium	17 (31%)	16 (14%)		33 (20%)
Low	21 (38%)	39 (36%)		62 (37%)
Relative poverty	14 (25%)	38 (35%)		52 (32%)
Extreme poverty	2 (4%)	6 (6%)		8 (5%)
Annual dental prophylaxis	29 (54%)	42 (39%)	0.066	71 (44%)
Median ESR (*P*_25_;*P*_75_) (mm/h)	19.5 (9.8; 32.0)	26.0 (16.5; 36.5)	0.017	24.0 (12.0; 34.0)
Median CRP (*P*_25_;*P*_75_) (mg/L)	2.8 (1.2; 5.0)	3.4 (1.62; 7.3)	0.077	3.4 (1.4; 6.2)
Bone mineral density			0.624	
Normal	21 (48%)	34 (41%)		55 (43%)
Osteopenia	15 (34%)	28 (34%)		43 (34%)
Osteoporosis	8 (18%)	21 (25%)		29 (23%)
Tobacco			0.709	
Never	30 (54%)	59 (54%)		89 (54%)
Former smoker	13 (24%)	31 (28%)		44 (27%)
Current	12 (22%)	19 (17%)		31 (19%)
Stress	9 (17%)	36 (33%)	0.028	45 (28%)
Diabetes (total)	10 (18%)	8 (7%)	0.036	18 (11%)
Type II	9 (16%)	6 (5%)	0.074	15 (9%)
Dyslipidemia	27 (49%)	56 (51%)	0.318	83 (50%)
Hypercholesterolemia	12 (22%)	31 (28%)		43 (26%)
Hypertriglyceridemia	4 (7%)	13 (12%)		17 (10%)
Mixed hyperlipidemia	11 (20%)	12 (11%)		23 (14%)
Hypertension	18 (33%)	33 (30%)	0.749	51 (31%)
Myocardial infarction	2 (4%)	5 (5%)	0.776	7 (4%)
Mean BMI (SD) (kg/m^2^)	27.1 (3.69)	28.1 (5.20)	0.174	27.8 (4.8)
Categories			0.026	
Normal, 18.5–24.99	14 (25%)	33 (30%)		47 (29%)
Overweight, 25–29.99	30 (55%)	38 (35%)		68 (41%)
Obesity I, 30–34.99	11 (20%)	28 (26%)		39 (24%)
Obesity II, ≥ 35	–	10 (9%)		10 (6%)

Data represent numbers, percentages, mean (SD), or median (*P*_25_; *P*_75_)

*Anti-CCP* anti-cyclic citrullinated peptide, *BMI* body mass index, *CRP* C-reactive protein, *ESR*, erythrocyte sedimentation rate, *RA* rheumatoid arthritis, *SD* standard deviation

Table 2 depicts the disease characteristics in patients with positive and negative anti-CCP antibodies. There were statistical differences between anti-CCP antibody negative and positive patients in terms of RF seropositivity (46% vs 87%, *p* <  0.001), RF titers (63 ± 90.48 vs 234.5 ± 392.73, *p* <  0.001), DAS28 (ESR) (3.47 ± 1.31 vs 3.98 ± 1.34, *p* = 0.023), and SDAI (12.16 ± 8.97 vs 15.77 ± 11.77, *p* = 0.043). The same tendency was observed when disease activity was assessed by DAS28 (CRP) (2.95 ± 1.15 vs 3.30 ± 1.22, *p* = 0.069). However, when patients were categorized by disease activity (remission, low, moderate, or high activity), no significant differences were observed between anti-CCP antibodies positive and negative patients. Nor were any differences noted between the two groups with respect to treatment with glucocorticoids, sDMARDs, or bDMARDs.

**Table 2:** Disease, activity, and periodontal characteristics with respect to anti-CCP antibodies

	Anti-CCP negative (*N* = 55)	Anti-CCP positive (*N* = 109)	*p* value	Total (*N* = 164)
Median time evolution (*P*_25_;*P*_75_) years	7.3 (2.0; 12.0)	6.6 (2.5; 11.8)	0.852	6.8 (2.4; 11.9)
Early RA (less than 2 years)	14 (26%)	23 (21%)	0.529	37 (23%)
Established RA	41 (75%)	88 (81%)	0.361	129 (79%)
Rheumatoid factor				
Seropositive	25 (46%)	95 (87%)	< 0.001	120 (73%)
RF titers (I.U./mL)	63.0 (90.48)	234.5 (392.73)	< 0.001	177.33 (334.32)
Low (< 90)	38 (72%)	43 (41%)		81 (51%)
Moderate (91–300)	14 (26%)	40 (38%)		54 (34%)
High (> 300)	1 (2%)	23 (22%)		24 (15%)
Level of activity				
DAS28 (SD)	3.47 (1.31)	3.98 (1.34)	0.023	3.81 (1.35)
DAS28-CRP (SD)	2.95 (1.15)	3.30 (1.22)	0.069	3.19 (1.21)
SDAI (SD)	12.16 (8.97)	15.77 (11.63)	0.045	14.56 (10.92)
CDAI (SD)	10.92 (10.37)	13.65 (10.51)	0.116	12.73 (10.51)
Disease activity categories			0.122	
Remission	16 (29%)	20 (17%)		34 (21%)
Low	12 (22%)	21 (19%)		33 (20%)
Moderate	23 (42%)	51 (47%)		74 (45%)
High	4 (7%)	19 (17%)		23 (14%)
Glucocorticoid therapy			0.700	
No glucocorticoids	29 (53%)	54 (49%)		83 (51%)
Glucocorticoids	26 (47%)	55 (51%)		81 (49%)
Current dosage GC (SD) (mg/d)	2.84 (4.50)	3.19 (4.39)	0.636	3.07 (4.42)
Current dosage GC			0.239	
Low < 7.5	49 (89%)	93 (85%)		142 (86%)
Moderate 7.5–20	4 (7%)	15 (14%)		19 (12%)
High > 20	2 (4%)	1 (1%)		3 (2%)
Type of RA therapy			0.577	
No treatment	4 (7%)	6 (6%)		10 (6%)
sDMARDs	25 (46%)	62 (57%)		87 (53%)
≥ 2 sDMARDs	19 (34%)	31 (28%)		50 (31%)
bDMARDs	7 (13%)	10 (9%)		17 (10%)
Periodontitis*			0.276	
Level 0	–	4 (4%)		4 (3%)
Level 1	31 (56%)	53 (49%)		84 (51%)
Level 2	24 (44%)	52 (48%)		76 (46%)
Periodontitis**			0.422	
No	–	2 (2%)		2 (1%)
Stage I	8 (15%)	18 (17%)		26 (16%)
Stage II	21 (38%)	33 (30%)		54 (33%)
Stage III	16 (29%)	25 (23%)		41 (25%)
Stage IV	10 (18%)	31 (28%)		41 (25%)
Stages III+IV	26 (47%)	56 (51%)	0.370	82 (50%)
Mean PI	22.4 (13.3)	3.01 (19.7)	0.001	28.1 (18.2)
Mean PPD	2.99 (0.48)	3.16 (0.70)	0.069	3.10 (0.64)
CAL	3.72 (0.85)	4.16 (1.43)	0.015	4.01 (1.28)
Tooth Loss***	5.89 (5.06)	6.47 (5.53)	0.516	6.27 (5.37)
N° PPD ≥ 5 mm	11.64 (11.02)	16.93 (19.63)	0.029	15.15 (17.37)
% PPD ≥ 5 mm	0.09 (0.09)	0.14 (0.16)	0.014	0.12 (0.15)
% BoP	0.58 (0.19)	0.65 (0.24)	0.055	0.63 (0.23)

Data represent numbers, percentages, mean (SD), or median (*P*_25_;*P*_75_)

*Anti-CCP* anti-cyclic citrullinated peptide, *bDMARD* biologic disease-modifying anti-rheumatic drugs, *CAL* clinical attachment level, *CDAI* Clinical Disease Activity Index, *CRP* C-reactive protein, *DAS28* 28-joint Disease Activity Score with ESR, *DAS28-CRP* 28-joint Disease Activity Score with CRP, *ESR* erythrocyte sedimentation rate, *GC* glucocorticoids, *N° PPD ≥ 5 mm* number of pockets ≥ 5 mm, *PI* plaque index, *PPD* probing pocket depth, *RA* rheumatoid arthritis, *RF* rheumatoid factor, *SDAI* simplified disease activity index, *SD* standard deviation, *sDMARDs* synthetic disease-modifying anti-rheumatic drugs, *% BoP* percentage of sites with bleeding on probing, *%PPD ≥ 5 mm*: percentage of pockets ≥ 5 mm

*Levels of periodontitis according to Tonetti’s classification (2005)

**Levels of periodontitis according to Tonetti’s classification (2018)

***Tooth Loss: number of missing teeth

### Association between the presence and levels of anti-CCP antibodies and periodontal status in RA patients

Using the 2005 definition [34], the association between periodontitis and the presence of anti-CCP antibodies was not significant (*p* = 0.276), with an adjusted OR of 1.228 (95% CI 0.628–2.40, *p* = 0.549). When the 2018 definition was used [35], although the percentage of patients with severe periodontitis (stage III+IV) was higher in positive, versus negative, anti-CCP antibody patients (51 vs 47%, respectively), the association between periodontitis and the presence of anti-CCP antibodies was also not significant (*p* = 0.370), with an adjusted OR of 1.222 (95% CI 0.628–2.379, *p* = 0.555); indeed, it proved quite similar to the 2005 [34] periodontitis case definition. Therefore, the 2018 classification [35] of periodontitis was used for the following comparisons.

When periodontal outcome parameters were used instead of case definitions, anti-CCP positive patients showed significantly higher CAL (4.16 ± 1.43 vs 3.72 ± 0.85, *p* = 0.015), higher numbers (16.93 ± 19.63 vs 11.64 ± 11.02, *p* = 0.029) and percentages of pockets ≥ 5 mm (0.14 ± 0.16 vs 0.09 ± 0.09, *p* = 0.014), and higher mean PI (31.0 ± 19.7 vs 22.4 ± 13.3, *p* <  0.001), compared to their anti-CCP antibody negative counterparts.

Regarding the severity of periodontal parameters vis-à-vis the presence of anti-CCP antibodies, a highly significant association (*p* = 0.007) was observed between the mean PI and anti-CCP positivity after resulting in an adjusted OR of 1.033 (95% CI, 1.009–1.058). The mean CAL also was significantly associated with anti-CCP positivity with an adjusted OR of 1.523 (95%CI 1.057–2.194, *p* = 0.024). In terms of the number of pockets ≥ 5 mm, a clear tendency for a positive association with anti-CCP positivity was demonstrated (adjusted OR of 1.024 95% CI 0.999–1.048, *p* = 0.065). After adjusting for confounders, BoP was not associated with the presence of anti-CCP antibodies. Using AUC-ROC curves, the cut-off points of mean PI (24%) and the number of pockets ≥ 5 mm (17) were established for a significant association with presence of anti-CCP antibodies after adjusting for confounders, being the respective adjusted ORs of 2.33 (95% CI 1.17–4.65, *p* = 0.017) and 2.03 (95% CI 1.01–4.10, *p* = 0.048).

**Fig. 1:**
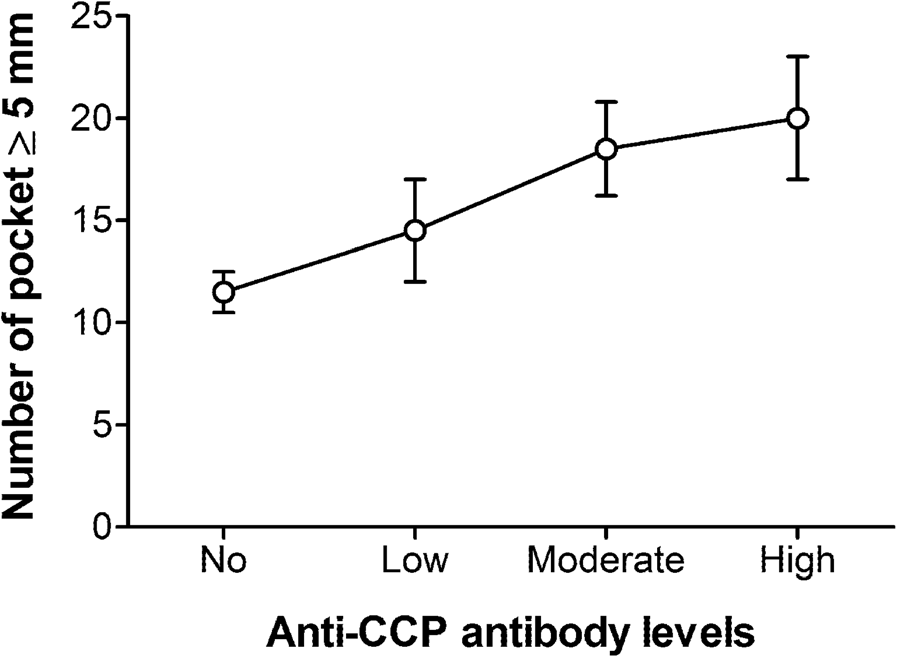
Number of pockets > 5 mm in RA patients negative or positive (categorized in low, moderate, or high titers) for anti-CCP antibodies. Data represent mean and SE of each group

When anti-CCP antibodies were stratified by level (low, moderate, and high), an ordinal logistic regression model showed a direct association between these levels and the mean CAL with an OR of 1.593 (95% CI 1.017–2.482, *p* = 0.043) in patients with high anti-CCP antibody titers versus non-anti-CCP RA patients (Table 3). Similarly, although with a more modest, albeit significant association was also demonstrated for patients with high anti-CCP antibody titers and the number of pockets ≥ 5 mm (Table 4, Fig. 1) and the mean PI (Table S1) with adjusted ORs of 1.031 (95%CI 1.003–1.062, *p* = 0.031) and 1.060 (95% CI 1.027–1.093, *p* < 0.001), respectively.

**Table 3:** Association between CAL and anti-CCP antibody levels (referred to its absence): ordinal logistic regression model

Anti-CCP levels	OR	95% conf. interval		*p*
Low				
CAL	1.227	0.742	2.027	0.425
Gender (ref. female)	1.267	0.385	4.166	0.697
Age	0.969	0.921	1.019	0.214
Tobacco (ref. never)	1.057	0.383	2.916	0.914
Disease activity (ref. remission/low)	1.115	0.403	3.084	0.834
Disease evolution time	0.956	0.883	1.036	0.277
Moderate				
CAL	1.657	1.101	2.509	0.017
Gender (ref. female)	1.589	0.552	4.573	0.390
Age	0.994	0.948	1.042	0.803
Tobacco (ref. never)	0.807	0.310	2.102	0.661
Disease activity (ref. remission/low)	1.601	0.621	4.126	0.330
Disease evolution time	1.012	0.952	1.076	0.705
High				
CAL	1.593	1.017	2.482	0.043
Gender (ref. female)	0.662	0.170	2.582	0.552
Age	0.971	0.923	1.021	0.243
Tobacco (ref. never)	0.850	0.305	2.369	0.756
Disease activity (ref. remission/low)	2.210	0.770	6.344	0.141
Disease evolution time	1.035	0.969	1.105	0.308

*Anti-CCP* anti-cyclic citrullinated peptide, *OR* odds ratio

**Table 4:** Association between the number of pockets ≥ 5 mm and anti-CCP antibody levels (referred to as its absence): ordinal logistic regression model

Anti-CCP levels	OR	95% conf. interval		*p*
Low				
No. of pockets ≥ 5 mm	1.010	0.977	1.043	0.560
Gender (ref. female)	1.337	0.410	4.357	0.630
Age	0.976	0.932	1.022	0.296
Tobacco (ref. never)	1.144	0.428	3.052	0.789
Disease activity (ref. remission/low)	1.196	0.442	3.231	0.725
Disease evolution time	0.957	0.883	1.037	0.279
Moderate				
No. of pockets ≥ 5 mm	1.022	0.996	1.050	0.087
Gender (ref. female)	1.699	0.604	4.777	0.315
Age	1.007	0.965	1.051	0.756
Tobacco (ref. never)	0.941	0.382	2.319	0.895
Disease activity (ref. remission/low)	1.745	0.709	4.297	0.226
Disease evolution time	1.012	0.953	1.075	0.689
High				
No. of pockets ≥ 5 mm	1.031	1.003	1.062	0.031
Gender (ref. female)	0.627	0.158	2.483	0.507
Age	0.983	0.938	1.030	0.471
Tobacco (ref. never)	0.898	0.331	2.436	0.832
Disease activity (ref. remission/low)	2.268	0.806	6.384	0.121
Disease evolution time	1.032	0.967	1.102	0.345

*Anti-CCP* anti-cyclic citrullinated peptide, *OR* odds ratio

Using a covariance analysis (ANCOVA), a significant increase of 4.45 U/mL (95% CI 1.60–7.29, *p* = 0.002) of anti-CCP titers was found for each pocket ≥ 5 mm in RA patients after adjusting for age, gender, smoking, time of disease evolution, and RA activity.

### Impact of tobacco consumption

Table 1 depicts the data on tobacco consumption and its possible association with anti-CCP titers. There were no significant differences (*p* = 0.709) between antibody-positive and antibody-negative RA patients with respect to smoking. Similarly periodontitis assessed by 2018 [35] Tonetti’s definition either independently or in combination with tobacco consumption showed a not-significant association with low, moderate, nor high titers of anti-CCP antibodies (see Supplementary Table S2). However, smoking was significantly associated with periodontitis severity (stages III or IV compared to stages 0, I, or II) in RA patients, with an adjusted OR of 2.085 (95% CI 1.100–3.951, *p* = 0.021 (Table 5)).

**Table 5:** Influence of tobacco consumption on periodontitis (stage III+IV vs others)

Variables	Coefficient	Standard error	OR	95% conf. interval		*p*
Age	0.034	0.017	1.035	1.000	1.071	0.051
Gender (ref. female)	0.305	0.429	1.357	0.585	3.147	0.477
Tobacco (ref. never)	0.735	0.326	2.085	1.100	3.951	0.021
Disease activity (ref. remission/low)	1.444	0.366	4.239	2.069	8.684	< 0.001
Disease evolution time	0.008	0.025	1.009	0.961	1.0589	0.733
Intercept	− 1.712	0.944	0.180			0.070

## Discussion

The most important findings of this study can be summarized as follows: (1) in RA patients, periodontal conditions such as the mean CAL, number of pockets ≥ 5 mm and PI were associated with the presence of anti-CCP antibodies; (2) this association was more pronounced in patients with higher levels of these antibodies; and (3) a linear smoking-independent correlation was found between periodontal subrogate parameters such as number of pockets ≥ 5 mm, and anti-CCP titers.

The presence of citrullinated proteins and ACPA in systemically healthy periodontitis patients has been reported in recent years [12, 24]. However, in RA patients the relationship between ACPA—specifically anti-CCP antibodies, the most commonly detected ACPA in clinics—and periodontitis has remained controversial. Although some studies have shown a link between ACPA and periodontitis in RA patients [17, 39], others did not, particularly when ACPA was assessed by the presence of anti-CCP antibodies [21, 24–26]. In this cross-sectional study, we have tried to clarify the plausible connection of periodontitis and anti-CCP antibodies in RA patients. Our results, in agreement with a previous study [10], showed that in RA patients, the presence of anti-CCP antibodies was not significantly associated with periodontitis (diagnosed according to Tonetti’s 2005 [34] and 2018 [35] case definitions) after adjusting for confounders. Conversely, when periodontal parameters assessing the severity of periodontitis were used, such as mean CAL and number of pockets ≥ 5 mm, there was a significant association with high anti-CCP titers, which suggests that the presence of specific periodontopathogens, such as *P. gingivalis* or *A. actinomycetemcomitans* in periodontitis cases, may plays a key role in the citrullination process and anti-CCP antibody formation, as hypothesized by some studies [10, 22, 40]. At the same time, recent evidence has put forward an association between periodontitis and *P. gingivalis* in anti-CCP+ pre-RA patients [41], suggesting that disease duration and activity may have an impact on the association between periodontitis and anti-CCP titers in RA patients. Nonetheless, our results have demonstrated that the associations between periodontitis severity parameters, such as CAL or pockets ≥ 5 mm, and anti-CCP titers existed independently of age, gender, smoking, time of disease evolution, and RA disease activity.

There is increasing evidence linking periodontitis to other non-anti-CCP ACPAs [24, 26]. In a group of 248 RA patients and 85 healthy controls, Lee et al. demonstrated a positive correlation between antibodies against human α-enolase (ENO1) and PPD, BoP, and CAL [26]. In this work, anti-ENO1 antibodies significantly correlated with anti-CCP antibody titers and RA parameters, such as DAS28 [26]. Further studies, including anti-CCP and anti-ENO1 antibodies, in addition to other types of ACPAs such as anti-fibrinogen, anti-vimentin, or anti-CEP-1 antibodies could help to clarify the role of periodontitis and periodontal pathogens, such as *P. gingivalis* and *A. actinomycetemcomitans* in ACPA formation in RA patients, as well as their relationship with rheumatoid clinical variables.

In regard to periodontal variables and anti-CCP antibody titers, there is some evidence that links BoP, PI, and PPD to various ACPAs, including anti-CCP antibodies [10, 25, 26]. Interestingly, Mikuls et al. [10] reported a higher incidence of pockets ≥ 5 mm in anti-CCP-positive patients than controls, which remained true independently of smoking habit. Our results have also shown significantly higher levels of mean CAL, numbers and percentages of pockets ≥ 5 mm, mean PI, and mean PPD, in anti-CCP antibody-positive versus antibody-negative patients. Using logistic regression analysis and after adjusting for confounders, we identified a gradient effect in the association between mean CAL, mean PI, and the number of pockets ≥ 5 mm with anti-CCP antibody titers. In fact, all those periodontal parameters reached statistical significance in patients with high anti-CCP antibody titers (> 300). In addition, using AUC-ROC curves, we established cut-off points for mean PI and the number of pockets ≥ 5 mm vis-à-vis risk for the presence of anti-CCP antibodies in RA patients. RA patients with more than 17 pockets ≥ 5 mm, a situation characteristic of severe periodontitis, or a mean PI of 24% or higher, a finding that denotes poor oral hygiene, doubles the likelihood of being anti-CCP positive than those with periodontal parameters below those levels. Thus, in regard to pockets, we have established a previously unreported linear relationship between the number of pockets ≥ 5 mm and the quantity of anti-CCP antibodies. After adjusting for age, gender, smoking habit, time of disease evolution, and RA disease activity, we found in our group of RA patients that for every additional pocket ≥ 5 mm, the concentration of anti-CCP antibodies increased by 4.45 U/ml in RA patients. This relationship could reflect the potential role of *P. gingivalis* and *A. actinomycetemcomitans* in protein citrullination and ACPA formation, due to the fact that these periodontal pathogens are likely present in pockets ≥ 5 mm [42–44]. However, adequate studies using microbiological data are needed to correlate the presence of such bacteria, ACPA titers, and RA clinical variables.

Apart from periodontitis and presence of key periodontal pathogens as sources of citrullination leading to ACPA formation [10, 13, 15, 23, 26, 40], smoking may also contribute to citrullination and ACPA formation [7, 45]. Likewise, tobacco use is also a risk factor for periodontitis in both healthy [46] and RA patients [47]. Some authors have hypothesized that the impact of smoking may be so strong that it may mask the effects of *P. gingivalis* and periodontitis on citrullination and ACPA formation in RA patients [48], although there is also data associating periodontitis and anti-CCP antibody titers, independently from tobacco consumption in non-RA patients [23]. Our results have shown that tobacco use was associated with periodontitis (according to Tonetti’s case definitions) in RA patients, and at the same time, subrogate parameters of periodontitis severity such as CAL or PPD ≥ 5 mm were significantly associated with anti-CCP antibody titers after adjusting by age, gender, and smoking. However, both tobacco and periodontitis, either independently or in combination, did not show an association with anti-CCP antibodies titers in our series of RA patients. These results may be explained by the lack of microbiological data in our study, due to the fact that *P. gingivalis* and *A. actinomycetemcomitans* concentrations may play significant roles in ACPA formation, independently of tobacco consumption [10, 22, 40].

There are some limitations in this study. The design of our study does not address causality and the lack of microbiological data does not allow for assessing the possible association between pathogens and anti-CCP titers. The correlation between periodontitis pathogens, including *P. gingivalis* and *A. actinomycetemcomitans* and anti-CCP antibodies, should be the focus of future research to elucidate their impact on the pathogenesis of RA, as has been previously mentioned. Future research, including interventional studies focused on the influence of periodontal status and the bacteria responsible for periodontitis on both the presence of anti-CCP antibodies and disease activity, would help us better understand the causal connections between periodontitis and RA.

## Conclusions

There is a link between anti-CCP antibody titers and periodontitis, in which worsening periodontal conditions, in terms of mean CAL, mean PI, and number of pockets > 5 mm, were statistically associated with both anti-CCP antibody positivity and higher levels of anti-CCP antibody titers. However, tobacco consumption and periodontitis (assessed by Tonetti’s definition) does not seem to be associated with levels of anti-CCP antibodies in our RA patient series.

## Supplementary information

**Additional file 1: Table S1.** Association between mean PI and anti-CCP antibody levels (referred to its absence): ordinal logistic regression model.

**Additional file 2: Table S2.** Association between tobacco and periodontitis with anti-CCP antibody levels (referred to its absence): ordinal logistic regression model.

## Data Availability

The datasets used and/or analyzed during the current study are available from the corresponding author, who has the ORCID identifier 0000-0002-4139-9295, on reasonable request.

## Abbreviations

ACPAs: Anti-citrullinated peptide antibodies
ANCOVA: Analysis of the covariates
anti-CCP: Anti-cyclic citrullinated peptide
AUC-ROC: Areas under ROC curves
bDMARD: Biologic disease-modifying antirheumatic drug
BMI: Body mass index
BoP: Bleeding of probing
CAL: Clinical attachment level
CDAI: Clinical Disease Activity Index
CRP: C-reactive protein
DAS28: 28-joint Disease Activity Score
DMARD: Disease-modifying antirheumatic drug
ERA: Early rheumatoid arthritis
ESR: Erythrocyte sedimentation rate
OD: Odds ratio
PAD: Peptidil arginine deiminase
PI: Plaque index
PPD: Proving pocket depth
PSS-14: Perceived Stress Scale
RA: Rheumatoid arthritis
RF: Rheumatoid factor
ROC: Receiver operating characteristic
SD: Standard deviation
sDMARD: Synthetic disease-modifying antirheumatic drug
