# Supplementary material for: Association between periodontitis and anti-citrullinated protein antibodies in rheumatoid arthritis patients: a cross-sectional study
Source: Arthritis Res Ther. 2020 Feb 13;22:27. doi: 10.1186/s13075-020-2121-6 (PMC7020577; doi:10.1186/s13075-020-2121-6)
Supplement: Supplementary file 1 — Additional file 1: Table S1. Association between mean PI and anti-CCP antibody levels (referred to its absence): ordinal logistic regression model. [file 13075_2020_2121_MOESM1_ESM.docx]

**Table S1**. Association between mean PI and anti-CCP antibody levels (referred to its absence): ordinal logistic regression model.

| **Anti-CCP levels** | **OR** | **[95% Conf. Interval]** | | **P** |
| --- | --- | --- | --- | --- |
| Low |  |  |  |  |
| Mean PI | 1.011 | 0.978 | 1.044 | 0.523 |
| Gender (Ref. female) | 1.266 | 0.372 | 4.311 | 0.706 |
| Age | 0.976 | 0.931 | 1.022 | 0.301 |
| Tobacco (Ref. never) | 1.210 | 0.460 | 3.179 | 0.699 |
| Disease activity (Ref. remission/low) | 1.150 | 0.413 | 3.197 | 0.789 |
| Disease evolution time | 0.960 | 0.887 | 1.039 | 0.316 |
| Moderate |  |  |  |  |
| Mean PI | 1.025 | 0.997 | 1.053 | 0.064 |
| Gender (Ref. female) | 1.530 | 0.524 | 4.467 | 0.436 |
| Age | 1.008 | 0.966 | 1.053 | 0.703 |
| Tobacco (Ref. never) | 1.057 | 0.437 | 2.559 | 0.902 |
| Disease activity (Ref. remission/low) | 1.666 | 0.660 | 4.203 | 0.280 |
| Disease evolution time | 1.017 | 0.958 | 1.079 | 0.586 |
| High |  |  |  |  |
| Mean PI | 1.060 | 1.027 | 1.093 | <0.001 |
| Gender (Ref. female) | 0.304 | 0.065 | 1.417 | 0.130 |
| Age | 0.991 | 0.943 | 1.040 | 0.704 |
| Tobacco (Ref. never) | 0.985 | 0.354 | 2.744 | 0.978 |
| Disease activity (Ref. remission/low) | 1.551 | 0.520 | 4.628 | 0.431 |
| Disease evolution time | 1.028 | 0.959 | 1.101 | 0.436 |
| Anti-CCP: Anti-cyclic citrullinates peptide; OR: odds ratio. | | | | |
