# Supplementary material for: Association between periodontitis and anti-citrullinated protein antibodies in rheumatoid arthritis patients: a cross-sectional study
Source: Arthritis Res Ther. 2020 Feb 13;22:27. doi: 10.1186/s13075-020-2121-6 (PMC7020577; doi:10.1186/s13075-020-2121-6)
Supplement: Supplementary file 2 — Additional file 2: Table S2. Association between tobacco and periodontitis with anti-CCP antibody levels (referred to its absence): ordinal logistic regression model. [file 13075_2020_2121_MOESM2_ESM.docx]

**Table S2**. Association between tobacco and periodontitis with anti-CCP antibody levels (referred to its absence): ordinal logistic regression model.

| **Anti-CCP levels** | **OR** | **[95% Conf.** | **Interval]** | **P** |
| --- | --- | --- | --- | --- |
| Low |  |  |  |  |
| Periodontitis (Ref. stage 0+I+II) | 0.919 | 0.222 | 3.805 | 0.907 |
| Tobacco (Ref. never) | 2.307 | 0.633 | 8.404 | 0.205 |
| Periodontitis x Tobacco^*^ | 0.261 | 0.036 | 1.874 | 0.182 |
| Gender (Ref. female) | 1.492 | 0.446 | 4.986 | 0.516 |
| Age | 0.981 | 0.936 | 1.029 | 0.431 |
| Disease Activity (Ref. remission/low) | 1.465 | 0.514 | 4.176 | 0.475 |
| Disease evolution time | 0.957 | 0.882 | 1.039 | 0.296 |
| Moderate |  |  |  |  |
| Periodontitis (Ref. stage 0+I+II) | 1.213 | 0.364 | 4.037 | 0.753 |
| Tobacco (Ref. never) | 1.167 | 0.303 | 4.499 | 0.822 |
| Periodontitis x Tobacco^*^ | 0.819 | 0.140 | 4.781 | 0.824 |
| Gender (Ref. female) | 1.838 | 0.668 | 5.056 | 0.239 |
| Age | 1.006 | 0.964 | 1.051 | 0.777 |
| Disease Activity (Ref. remission/low) | 1.906 | 0.758 | 4.792 | 0.170 |
| Disease evolution time | 1.017 | 0.958 | 1.079 | 0.587 |
| High |  |  |  |  |
| Periodontitis (Ref. stage 0+I+II) | 1.147 | 0.307 | 4.287 | 0.839 |
| Tobacco (Ref. never) | 1.020 | 0.244 | 4.643 | 0.980 |
| Periodontitis x Tobacco^*^ | 1.033 | 0.144 | 7.400 | 0.974 |
| Gender (Ref. female) | 0.732 | 0.195 | 2.748 | 0.644 |
| Age | 0.983 | 0.937 | 1.030 | 0.464 |
| Disease Activity (Ref. remission/low) | 2.628 | 0.912 | 7.575 | 0.074 |
| Disease evolution time | 1.038 | 0.973 | 1.107 | 0.256 |
| Anti-CCP: Anti-cyclic citrullinates peptide; OR: odds ratio.  * Interaction between tobacco and periodontitis | | | | |
